# Bio-Inspired Miniature Direction Finding Acoustic Sensor

**DOI:** 10.1038/srep29957

**Published:** 2016-07-21

**Authors:** Daniel Wilmott, Fabio Alves, Gamani Karunasiri

**Affiliations:** 1 Department of Physics, Naval Postgraduate School, Monterey, CA 93943, USA.

## Abstract

A narrowband MEMS direction finding sensor has been developed based on the mechanically coupled ears of the *Ormia Ochracea* fly. The sensor consists of two wings coupled at the middle and attached to a substrate using two legs. The sensor operates at its bending resonance frequency and has cosine directional characteristics similar to that of a pressure gradient microphone. Thus, the directional response of the sensor is symmetric about the normal axis making the determination of the direction ambiguous. To overcome this shortcoming two sensors were assembled with a canted angle similar to that employed in radar bearing locators. The outputs of two sensors were processed together allowing direction finding with no requirement of knowing the incident sound pressure level. At the bending resonant frequency of the sensors (1.69 kHz) an output voltage of about 25 V/Pa was measured. The angle uncertainty of the bearing of sound ranged from less than 0.3° close to the normal axis (0°) to 3.4° at the limits of coverage (±60°) based on the 30° canted angle used. These findings indicate the great potential to use dual MEMS direction finding sensor assemblies to locate sound sources with high accuracy.

In 1776, the physicist J. B. Venturi, most widely known for his work in fluid dynamics, surmised that the ability to find the direction of a sound source was based on the amplitude difference between two ears^1^. Lord Rayleigh confirmed this in 1907 by concluding that the different distances sound travelled to each ear resulted in a phase difference, analogous to an amplitude difference for periodic sound waves^2^. In animals with a relatively large ear separation compared to sound wavelength the delay of the sound arrival, inter-aural time difference (ITD), and variation in the pressure field between ears, inter-aural level difference (ILD), allows for direction finding (DF). Humans use this principle to determine sound direction with up to 2 degrees accuracy^3^. However, there are insects such as the parasitic fly *Ormia Ochracea* with much smaller separation of ears have developed unique approach to direction finding^4^. The female of this species seek out chirping crickets to lay their eggs on, and do so with an accuracy of less than 2 degrees^4^. The two eardrums of the fly are separated by a mere 0.5 mm yet it homes in on the cricket chirping with a 7 cm wavelength, using an ITD of at most 2.5 μs and negligible ILD^4^.

Miles *et al.*^4^ found that the two ear drums of the fly are mechanically coupled and have two natural resonant frequencies. In the first mode the ears move out of phase with each other in a pure rocking mode due to the minute pressure difference between the two eardrums. The second mode has the eardrums moving in phase, resulting in a pure bending mode about the tympanal bridge. The bending mode is a result of the sum of the forces on the eardrums. These modes, caused by the mechanical link between eardrums, give the *Ormia Ochracea* “remarkable sensitivity to the direction of an incident sound stimulus”^4^. The fly employs the coupling between the two modes at the chirp frequency of cricket to sense the direction making use of the unequal vibrational amplitudes of the two eardrums^4^. It is reasonable to expect that the bending mode (Fig. 1(a)) produces a greater displacement of the ear drums as they experience the full force of the sound pressure whereas the rocking mode (Fig. 1(a)) is due to the difference in sound pressure. There is a number of reports^5^,^6^,^7^,^8^,^9^,^10^,^11^,^12^,^13^,^14^,^15^,^16^,^17^ on attempts to develop miniature sensors based on the fly’s hearing system with different degree of success. The common feature of these approaches is that the sensors were primarily operated at resonant frequency of a mechanical system oppose to off resonance detection employed in conventional broadband microphones. The response of these sensors to sound was probed optically by reflecting light from the vibrating structures^8^,^9^,^13^ or electronically using either integrated comb finger capacitors^10^,^14^,^17^ or integrated piezoelectric pads^15^,^16^.

The motivation focus of this research is for localization of sound sources with sensors much smaller than the wavelength they detect, making MEMS *Ormia*-based detectors very attractive. In an earlier publication^14^ individual sensors operating at the bending mode were demonstrated with symmetric directional response around the normal incidence making the determination of bearing ambiguous. In addition, the knowledge of pressure at the sensor is needed since the sensor response depends both on the angle of arrival and pressure of the sound. In this paper, we report on an unambiguous directional sensing approach using two canted sensors where the knowledge of the sound pressure is no longer required.

## Results

**Figure 1:**
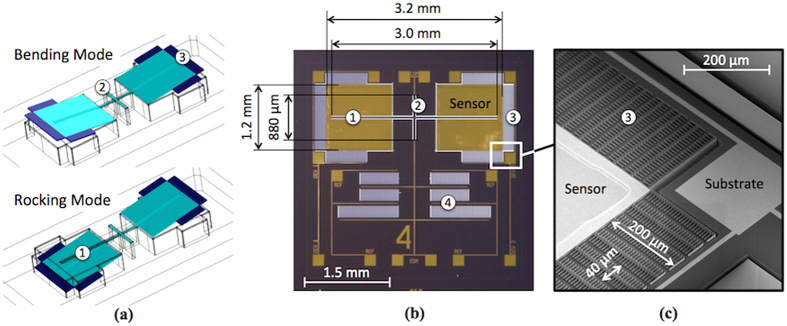
MEMS directional acoustic sensor. (**a**) Simulated displacements of the sensor showing bending and rocking modes of oscillation. (**b**) Optical micrograph of the fabricated sensor showing the bridge that connects two wings (1), the torsional legs that connect the free standing sensor to the substrate (2), the interdigitated comb finger capacitors (3) and the reference capacitors made of fixed electrodes (4). (**c**) SEM image of section of the sensor with comb finger capacitors.

The sensor reported in this paper, designed to operate around 1.7 kHz, consists of two 1.2 × 1.2 mm^2^ wings connected in the middle by a 3 mm × 30 μm bridge. The entire structure is connected to the substrate by two torsional legs at the center. Figure 1(b) shows the michroelectromechanical system (MEMS) sensor, which was fabricated by MEMSCAP, a commercial foundry specializing in Silicon-on-Insulator Multi User Manufacturing Process (SOIMUMPS)^18^. In their process the SOI substrate thickness is 400 μm with a 25 μm thick device layer and etching is available on both sides of the wafer that results in a 25 μm thick device with a trench on the back. For electronic readout of nanoscale vibration amplitudes at typical sound pressures, a set of interdigitated comb finger capacitors was integrated at the edges of the wings^10^,^14^ (Fig. 1(b,c), identified by the label (3)). The comb fingers are designed in a fishbone architecture with a 200 μm long central spine with 20 μm long and 2 μm wide comb fingers on both sides. The gap between moving fingers attached to the wings and fixed fingers attached to the substrate is 2 μm. Using the dimensions of the comb finger capacitors, the total capacitance was mathematically estimated to be about 20 pF. The SEM image in Fig. 1(c) indicates that the combs are mostly aligned with a slight tilt due to residual stress^14^. In addition to the comb finger capacitors attached to the wings, a reference capacitor made of fixed electrodes (Fig. 1(b), label (4)) with the same size was fabricated next to the sensor to allow differential measurement of the displacement using a MS3100 chip from the Irvine Sensors^19^. The sensor is operated at the bending resonance frequency (Fig. 1(a)) due to its larger amplitude of vibration.

**Figure 2:**
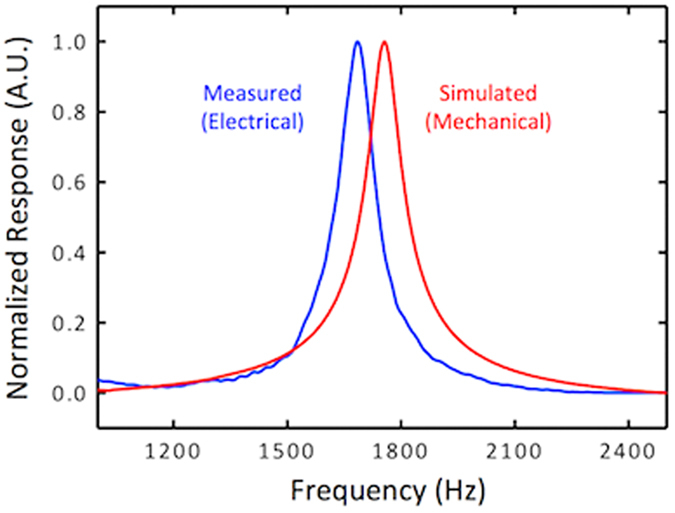
Measured and simulated frequency responses of a single sensor. The experimental response was measured using the electrical output generated by the comb finger capacitors, while the mechanical simulated response was obtained using COMSOL Multiphysics. The bending resonant frequency was found to be around 1690 Hz.

Figure 2 shows a comparison of measured and simulated frequency responses of a sensor. The simulation was carried out using COMSOL Multiphysics software with incident sound wave coupled to the MEMS sensor via acoustic-structure interaction. The damping was incorporated in the simulation using Couette flow generated damping force (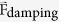) between moving and fixed geometrics of the sensor using^20^


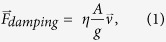


where η is the viscosity, A is the interacting surface area of the fixed and moving geometries, *g* is the gap between the moving and fixed surfaces and 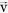 is the velocity. The acoustic waves were incorporated in the model using Background Pressure Field option, available in the Pressure Acoustic Module in COMSOL. The damping was incorporated as a Boundary Load along the thickness of the combs within the Solid Mechanics Module. The simulated mechanical response of the sensor as a function of frequency is shown in Fig. 2. The displacement, which is nearly the same for both wings, was normalized to allow comparison with the measured electrical response. It can be seen in Fig. 2 that the measured and simulated bandwidth of the sensor, full width at half maxima (FWHM), is about 125 Hz and are in close agreement without using any adjustable parameters in estimating the damping. The slight difference (<5%) between the measured and simulated resonance frequencies is most likely due to the use of designed dimensions and bulk material properties in the simulation. The measured bending resonant frequency of the sensor is at about 1690 Hz, which primarily depends on the dimensions of the bridge in the middle and mass of the wings.

**Figure 3:**
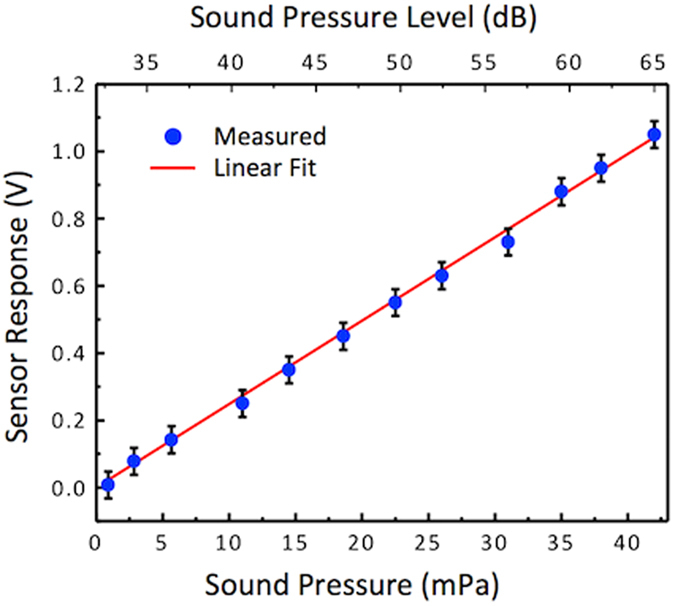
Measured electrical output at normal incidence (θ = 0°) with varying sound pressure of a single sensor. The resulting sensitivity of sensor is estimated to be about 25 V/Pa using a linear fit line.

Using sound incident normal to the sensor wings (θ = 0°) to elicit maximum output, the response of a sensor was measured by varying sound pressure as shown in Fig. 3. During the measurement, the sound frequency was set to 1690 Hz. The data in Fig. 3 shows that the response has a linear dependence to sound pressure and the slope of the line gives sensitivity of about 25 V/Pa. This value was obtained at the bending frequency, measured directly at output of the MS3110 readout chip. The readout chip was programed using a feedback capacitance (C_F_) of 1.06 pF and an internal gain setting of 4 which give sensitivity of about 10 V/pF based on the formula given in the MS3110 manual^19^. No external amplifiers were used.

**Figure 4:**
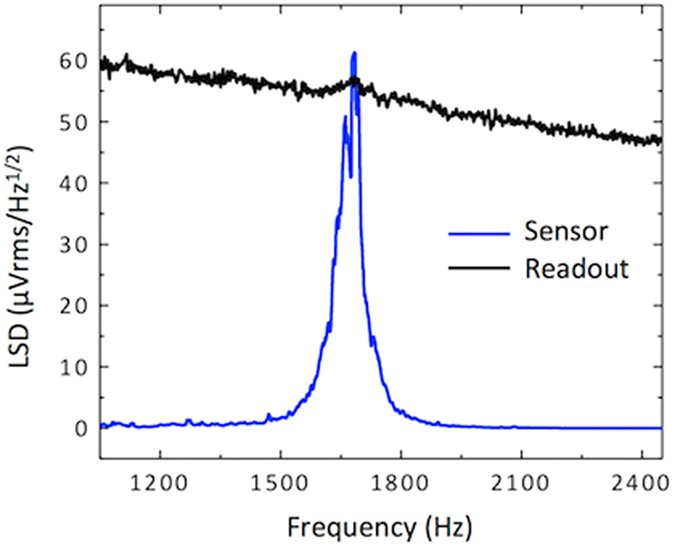
Measured electrical and mechanical noise of the sensor. The black solid line is the electrical linear spectral density at the output of the electrical readout for a sensor inside the anechoic chamber without any sound stimulus. The blue solid line represents the mechanical noise measured by a laser vibrometer, translated to voltage spectral density. Note the electrical noise is higher than the mechanical noise except near the resonant frequency of the sensor.

The intrinsic mechanical noise of the sensor is estimated to be about 11 dB primarily due to the vibration of the wings as a result of thermal agitations via surrounding air. The vibration amplitude as a function of sound frequency was measured using a laser vibrometer without external sound excitation, exhibiting a maximum of around 18 pm at the bending frequency. The peak mechanical sensitivity of the sensor was found to be about 25 μm/Pa. The amplitude of vibration was converted to linear spectral density and subsequently multiplied by the sensitivity of the sensor to translate the mechanical noise of the sensor to an equivalent electrical output. The combined electrical noise of the sensor and readout electronics was also measured in the same frequency range using a HP 3562A dynamic signal analyzer and the two voltage spectral densities are shown in Fig. 4. It can be seen in Fig. 4 that the electrical noise is dominant except at the resonance frequency of the sensor.

**Figure 5:**
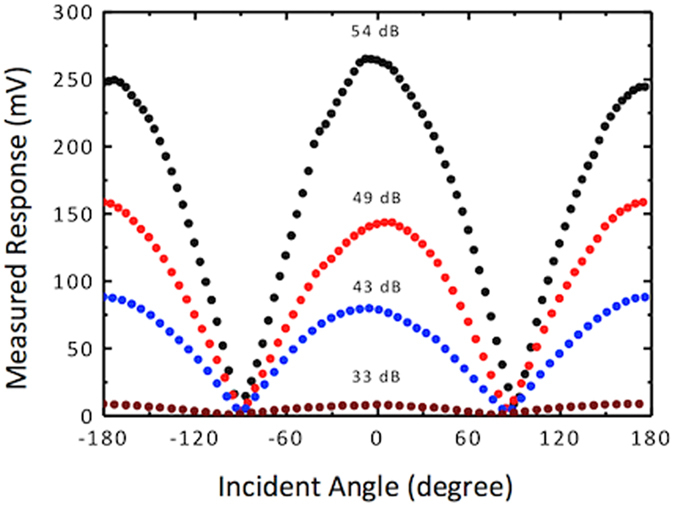
Measured directional responses of a single sensor at different sound levels. The measured electrical output follows a cosine dependence that can be used to determine incident angle from a given sensor output. An angle ambiguity exists on either side of 0° (normal incidence) where the same sensor output occurs for two angles mirrored about 0°.

Since the sound interacts with both sides of our MEMS sensor, it acts as a pressure gradient microphone with expected cosine dependence of the amplitude of vibration with direction of sound^8^. If the incident sound pressure amplitude at the sensor is P_o_, then the output voltage (V) as a function of incident angle has the form^10^


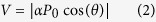


where α is a proportionality constant that depends on the parameters of the readout circuit and θ is the direction of arrival with respected to the normal. The output signal of the sensor was measured as the incident angle is varied from −180° to +180° for a set of sound pressures and the results are shown in Fig. 5, which agrees well with the expected cosine dependence given in equation (2). The directional response was observed for sound levels at the sensor down to 33 dB, which is close to the sound floor of the anechoic chamber used in the experiment. This indicates the high sensitivity of the comb finger electronic readout system.

**Figure 6:**
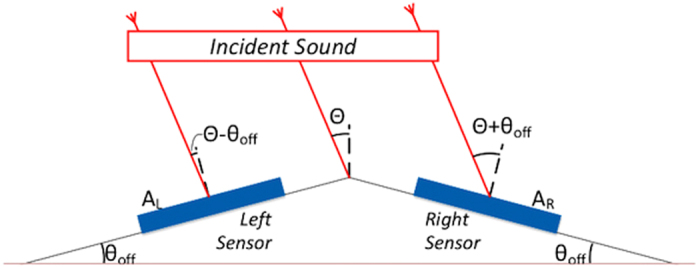
Arrangement of multiple MEMS DF sensors. In this arrangement two sensors are co-located at an angle θ_off_ such that the incident sound will interact at θ − θ_off_ at the left sensor and θ + θ_off_ at the right sensor. This will provide an effective coverage of θ from −90 + θ_off_ to +90 − θ_off_ with no angle ambiguity or requirement to measure the incoming sound level.

A single DF sound sensor performs adequately to provide the direction of sound (θ) in 0 to 90° range, however as shown in Fig. 5, there is an ambiguous angle result at −θ due to the symmetry of the response. In addition, to properly determine the angle using equation (2) the sound pressure at the sensor, *P*_*o*_ must be known requiring the use of a calibrated omnidirectional microphone. However, use of such a microphone will substantially reduce the sensitivity due to their operation in off resonance. To eliminate these requirements, two DF sensors can be arranged at a canted angle as per Fig. 6 similar to that used by the radar community for determining the target bearing using monopoles^21^,^22^.

Because each sensor produces an output (V) with cosine dependence as in equation (2) and both are symmetrically positioned at an offset angle θ_off_, the angle ambiguity can be solved. Both sensors are co-located in close proximity to each other, such that the amplitude of sound pressure, P_o_ can be considered nearly the same at both sensors. Applying equation (2) to the left (index L) and right (index R) sensors, the signal generated by the two sensors can be written as:


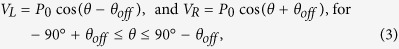


where α_L_ and α_R_ are calibration constants, which accounts on any mismatch between sensors and can be obtained by measuring the output of each sensor keeping sound pressure and incident angle the same. Taking the ratio of the difference and sum of normalized signals in equation (3), the unknown sound pressure amplitude can be eliminated to obtain the unknown angle using .


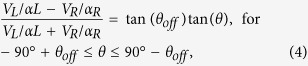


Using the measured electrical outputs of the two sensors (V_L_ and V_R_) and the corresponding proportionality constants (V_L_ and V_R_), the unknown angle can be readily obtained using equation (4). Because the sensor output is a measure of the magnitude of wing displacement, equation (4) is only valid within the specified range of angles as indicated.

**Figure 7:**
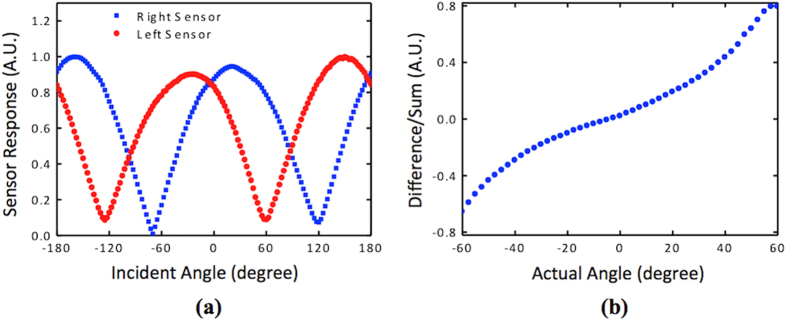
Directional response of the individual and combined acoustic sensors. (**a**) Normalized sensor outputs as a function of incident angle of sound and (**b**) difference over sum of the data showed in (**a**).

Two sensors canted at a 30° offset angle were employed for determining their ability to uniquely determine the incident angle of sound. The selection of 30° offset angle was made to obtain a relatively wide angular range while keeping the difference/sum ratio at an appreciable range based on Eqn. 4. It can be seen that smaller the canted angle wider the angular range but smaller the ratio due to nearly equal incident angles at the two sensors. Initially, angular dependence output of both sensors were measured for a sound pressure of 42 dB to determine the two proportionality constants (α_L_ and α_R_). Figure 7(a) shows the measured normalized responses of the two sensors as a function of the incident angle of sound from −180° to +180°. The two responses are as expected shifted from each other by about 60 degrees due to the use of θ_off_ = 30°. The fact that the signals from the sensors do not always cross zero is most likely due to the detection of scattered sound from the fixtures used in mounting the sensor assembly. Figure 7(b) shows the difference over sum ratio of the two normalized amplitudes for the range from −60 to +60° where equation (4) is valid and which serves as the calibration curve for the two-sensor assembly. There, the data were not averaged, nevertheless they were directly derived from the curves provided in Fig. 6(a).

**Figure 8:**
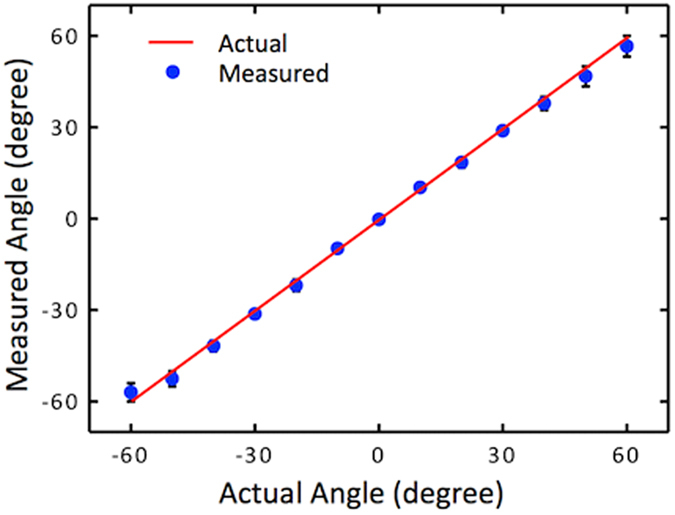
Comparison between actual and sensed angle of arrival in the canted sensors assembly. Average angle measurement using 33–54 dB source levels. Maximum error of ±3.4° occurred at the widest angles near ±60°.

Next, measurements were taken at 10° intervals over the range of ±60° for a set of sound pressure levels (33, 35, 37.5, 42, 49 and 54 dB). It was found that the ratio of difference and sum of the normalized amplitudes hardly varied with the sound pressure due to the linearity of the sensor response with pressure (see Fig. 3). This indicates that the dual sensor assembly does not require a sound level measurement to determine the direction of incident sound. For comparison of measured with the actual at each of the angles, measured output of the sensors at the six sound levels were averaged and ratio of normalized difference over sum was used to determine the measured angle. Figure 8 shows a comparison between measured and actual angles along with an ideal response line that corresponds to a 45° slope. Error bars represent the difference between six measurements taken at each angle, with minimal error close to the normal axis and maximum error of 3.4° as the angle of incidence increased to ±60°. Higher variation at larger angles is probably due to rapid increase of the ratio as the incident angle is increased making the determination of the angle less accurate. However, overall accuracy of determination of the direction is close to that of the fly’s hearing system^4^.

## Discussion

A MEMS direction finding sensor has been developed based on the mechanically coupled ears of the *Ormia Ochracea* fly. The operation of a single sensor at bending mode (1.69 kHz) produced a 25 V/Pa output. Two sensors that were co-located at an angle were used for determining the direction of sound. After calibration, the difference of the sensor outputs is divided by the sum to eliminate the unknown sound pressure. This resulted in an output that follows a tangent dependence, with a unique output for each angle across a 120° range employed in the measurement. It was found that the measured and the actual angles agree well with uncertainty of less than about 4° within the entire sound pressure range. There were many variations of sensors reported to mimic the fly’s hearing system. Most of them were operated at rocking motion of a mechanical structure to achieve the directional sensing. This led to relatively small signal (∼0.3 V/Pa)^17^ since the rocking motion depends on the minute pressure gradient along the sensor. The sensors employed in this work operated at the bending resonance, which provided high sensitivity (∼25 V/Pa) since the bending occurs due to the pressure of the incident sound wave. The directional response was obtained by keeping the backside of the sensor is open which generates a pressure gradient across the sensor^10^. The demonstrated accuracy and unambiguous measurement of the sound direction without the knowledge of the sound pressure, indicates a great potential to use this MEMS direction finding sensor for localization of sound sources having frequency component overlaps with that of the sensor. Future work includes the testing of the sensor performance in outdoors environment to determine its performance.

## Methods

**Figure 9:**
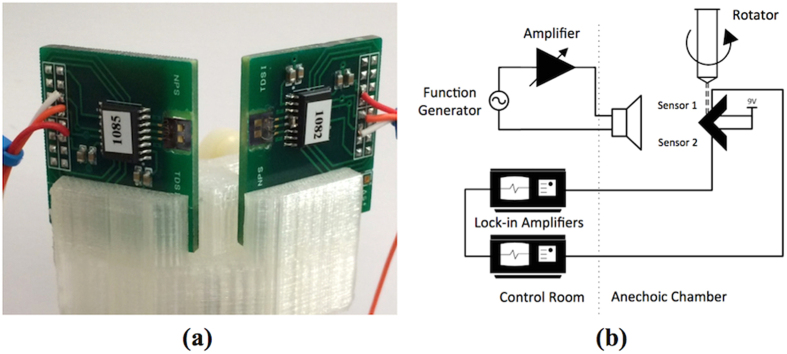
Canted sensors characterization method. (**a**) Fabricated dual sensor assembly and (**b**) schematics of the experimental setup used for the measurement of responses of the two sensors with angle and sound pressure.

The frequency response of the sensors was individually measured in an anechoic chamber by feeding the electrical output of the MS3110 chip to a lock-in amplifier. In order for the MS3110 to properly react to changes in capacitance at the sensor, it must be balanced using the built-in internal capacitors. The desired gain is set according to the expected capacitance variations and intended sound level. For our experiments the MS3110 was set to provide approximately 10 V/pF, where a pF corresponds a displacement of about 1 μm at the extremity of the sensor wing. The lock-in amplifier was a Stanford Research System model SR 850DSP and it was set to lock in the frequency of the sound source. An Agilent 33220A function generator was connected to an HP 467A audio amplifier to allow control of the speaker Selenium DH 200E used as a sound source. The sound level was measured by a Brüel & Kjaer 2670 pressure field microphone. The instrumentation was placed outside the anechoic chamber (control room, highlighted in Fig. 9(b)). The sensors were placed on 3D printed (Polylactic Acid (PLA)) mount, to assure 30 degree offset, as shown in Fig. 9(a). During the measurement, the two circuit boards were placed very close to each other and the separation of the two sensors was about couple of millimeters. The mount was connected to a metallic post connected to the turntable. All the wires passed through the turntable connection fixture.

The frequency of the excitation sound source was swept slowly to maintain the lock-in condition at all times. The sensor assembly was mounted on a remote controlled rotator 5 m away and at the same height as the speaker used for excitation. The sound was set to the desired levels while two lock-in amplifiers, one per each sensor channel, were used to capture the sensor output corresponds to excitation frequency of 1690 Hz.

The electrical noise measurements were performed connecting the output of the MS3110 chip to a HP 3652A dynamic signal analyzer. The sensor was kept inside our anechoic chamber during the measurement. The analyzer was set to provide the voltage spectral density for a frequency span of 800 Hz around the resonant frequency of the sensor. The measurement was repeated 100 times and averaged to provide the data shown in Fig. 4. The mechanical noise was measured using a Politec OFV-5000 laser vibrometer in the same frequency range.

## Additional Information

**How to cite this article**: Wilmott, D. *et al.* Bio-Inspired Miniature Direction Finding Acoustic Sensor. *Sci. Rep.* **6**, 29957; doi: 10.1038/srep29957 (2016).
